# Strengths and weaknesses of eye care services in sub-Saharan Africa: a meta-synthesis of eye health system assessments

**DOI:** 10.1186/s12913-020-05279-2

**Published:** 2020-05-06

**Authors:** Stevens Bechange, Emma Jolley, Bhavisha Virendrakumar, Vladimir Pente, Juliet Milgate, Elena Schmidt

**Affiliations:** 1 Sightsavers, Uganda Country Office, EADB Building, 4 Nile Avenue, Kampala, Uganda; 2 Sightsavers, Haywards Heath, UK; 3 Sightsavers, Cameroon Country Office, Yaoundé, Cameroon

**Keywords:** Health systems, Meta-synthesis, Eye care services, Eye heath system assessments, Sub-Saharan Africa

## Abstract

**Background:**

In sub-Saharan Africa (SSA), the delivery of eye care services continues to be undermined by health systems performance bottlenecks. There is a growing focus by partners in the sector on the analysis of the different components of eye care within the wider health system context to diagnose and manage interactions in ways that achieve more effective improvements. However, there has been no attempt to date to systematically synthesize these studies. In this study, we conducted a meta-synthesis of eye health system assessments to gain a more comprehensive understanding of the current systems and how they can be strengthened across different SSA contexts.

**Methods:**

We conducted a comprehensive search for eye health system assessment reports using global and regional websites of the WHO and other organizations supporting eye care in sub-Saharan Africa. A range of online databases with no language restrictions (PubMed, EMBASE, MEDLINE, PsycINFO and CINAHL) were searched for peer-reviewed publications referring to eye health system assessment (EHSA) or eye care service assessment tool (ECSAT). Assessments were included if they used the ECSAT or EHSA tool; were conducted in sub-Saharan Africa; and had been completed with full reports available in the public domain by January 15, 2019. A combination of framework and thematic syntheses was used.

**Results:**

Our search strategies yielded a total of 12 assessments conducted in nine countries using the ECSAT/EHSA tool in Sub-Saharan Africa**.** Eight assessments met our inclusion criteria: four were from West Africa, two from East Africa and two from Southern Africa. Across the eight countries, findings show considerable progress and improvements in the areas of governance, organisation, financing, provision, and coverage of eye care. However, several systemwide weaknesses were found to continue to impede quality eye health service planning and delivery across the countries included in this review.

**Conclusions:**

These findings highlight the need for national governments and iNGOs to invest in conducting and wider use of these assessments. Such analyses are particularly useful in building links between different system elements and in finding innovative, more flexible solutions and partnerships – needed to address avoidable vision loss in resource poor settings.

## Background

Governments across the world face diverse challenges in meeting their populations’ health needs [1]. This is especially the case in sub-Saharan Africa, where, despite significant reductions in infant and maternal mortality in recent decades, the burden of both communicable and non-communicable diseases remains high [2, 3]. There is an increasing global consensus that to be able to address the multiple challenges faced by some of the poorest countries in the world, “a health system strengthening” approach is required [4–6].

The World Health Organization (WHO) emphasized the importance of strengthening health systems in its 2007 framework for action [7] and in a range of other more recent publications and tools [8, 9]. The health system strengthening focus is also commonly acknowledged as an essential pre-requisite for achieving Universal Health Coverage, which has become a major global priority and a goal for health reforms in many Member States [10].

With 36 million people being blind and another 217 million living with moderate or severe visual impairment, eye health continues to be a global health problem, particularly in low income countries, where the delivery of eye care services is undermined by the scarcity of infrastructure, shortage of human resources and high user fees [11–15]. The WHO Global eye health action plan 2014–2019 aims to reduce avoidable visual impairment and secure access to rehabilitation services for the visually impaired and is built on the health system approach, which encompasses the integration of eye care services into the broader health systems at all levels [16].

With the understanding that the effectiveness of eye service delivery can only be improved through a better understanding and strengthening of eye health system functions, the WHO developed the Eye Care Service Assessment Tool (ECSAT), which provides guidance for assessing the status of a country’s eye care and helps decision-makers to identify gaps and strengthen access to and quality of services [17]. A few years earlier, a comparable tool, called the Eye Health System Assessment (EHSA) tool, was developed by a consortium of eye care and health experts coordinated by the International Centre for Eye Health (ICEH) at the London School of Hygiene and Tropical Medicine (LSHTM) with funding from Sightsavers [18]. Both tools are based on the six blocks of the WHO framework for strengthening health systems [7] and aim to identify strengths and weaknesses in eye care service provision within the wider health system context.

Completion of the ECSAT relies largely on desk review of publically available documents, reports, and other accessible secondary data sources. The EHSA tool on the other hand goes beyond desk review and incorporates the conduct of structured face to face interviews with several key actors at the Ministry of Health and other relevant government departments, professional societies, civil society organizations, relevant iNGOs and agencies working in-country. The in-depth nature of the questions that these respondents are usually asked allows for change on the different indicators to be probed and ascertained compared to previous years.

The purpose of this review is to synthesise data collected using the EHSA/ECSAT on governance, financing, provision and organisation of eye care services across different sub-Saharan Africa contexts, in order to gain a more comprehensive understanding of the current systems and how they can be strengthened to meet the objectives of the global eye health action plan and ultimtely better eye health for people in low resource settings.

## Methods

The review utilized the Enhancing Transparency in Reporting the Synthesis of Qualitative Research (ENTREQ) guidelines [19].

### Search strategy

The lead author (SB) conducted a comprehensive search of EHSA/ECSAT reports using global and regional websites of the WHO and other organizations supporting eye care in Sub-Saharan Africa. A range of electronic databases, including PubMed, MEDLINE, EMBASE, PsycINFO and CINAHL were searched for peer-reviewed publications referring to EHSA or ECSAT assessments. Search terms used included ‘eye health systems assessment’, ‘eye care service assessment tool’ and ‘sub-Saharan Africa’.

### Inclusion and exclusion criteria

The assessments were included in this review if they:

i) Used the ECSAT or EHSA tool.
ii) Were conducted in sub-Saharan Africa; and
iii) Had been completed with full reports available in the public domain by January 15, 2019.

There were no language limitations applied. The assessments that used tools other than the ECSAT or EHSA, were conducted outside Sub-Saharan Africa, those that were used for piloting the ECSAT before it was finalized and those that were incomplete at the time of this review were excluded.

### Study selection

Identified studies were screened for eligibility against the inclusion and exclusion criteria by the lead author (SB).

### Data extraction

Data from the included assessments were extracted independently by two authors (SB, VP) and checked between them for consistency. Where there was a disagreement a third author (ES) was involved to reach consensus. The full text of the reports was imported into NVIVO 11 qualitative analysis software [20] and coded. Appraisal was not undertaken because we had confidence in the relevance and conceptual richness of the primary assessments as we had been heavily involved in their conduct.

### Data synthesis

We used a meta-synthesis approach as it is a coherent approach to analysing data across qualitative studies. We used a combination of framework and thematic syntheses as our synthesis methodology [21], which involved three stages: coding text; development of descriptive themes; and generation of analytical themes (interpretation). Since all system assessments followed the WHO health system building blocks framework [7] to present the data, the framework was used as the first line of coding with subsequent codes assigned during the reading. This approach has been reported to have a number of advantages, particularly in situations when a large volume of evidence has to be synthesized over a short period of time to answer specific policy-relevant questions and still leaving opportunities for finding the ‘best fit’ in light of the evidence reported [21].

### Research team and reflexivity

All the authors of this paper are employees of Sightsavers. SB and VP are based in Uganda and Cameroon respectively while the others are based in the United Kingdom. ES provided technical support to the study team during the conduct of both the Ghana and Sierra Leone eye health systems assessments included in this review. SB, ES, VP, BV and EJ actively supported the conduct of the eye health systems assessments in Senegal, Mali, Kenya, Tanzania, Malawi, and Mozambique. The technical support requirements for the conduct of these eye health system assessments can be less or much greater in some countries, so our support varied from country to country depending on the expressed capacity gaps by the national eye care coordinator and the wider assessment team. JM oversees the policy and global advocacy function at Sightsavers and coordinates closely with relevant individuals at WHO on matters pertaining to eye health system assessments in the region.

### Ethical considerations

This review is based on secondary analysis of data available in the public domain and did not require ethics approval. All assessments except the Malawi ECSAT, which used routinely available data only, had received ethics approvals from their respective national Institutional Review Boards (IRB). The Ghana and Sierra Leone assessments also received ethics clearance from the research ethics committee at the LSHTM. All assessments received approvals from the national Ministries of Health.

## Results

### Search results

No peer-reviewed studies identified through our search met the inclusion criteria. Our search strategies returned a total of 12 assessments conducted in nine countries using the ECSAT/EHSA tool in Sub-Saharan Africa. Eight assessment reports fulfilled the inclusion criteria and were included in this meta-synthesis.

### Description of the included assessments

A total of eight assessments were included in this review: four were from West Africa (Ghana, Sierra Leone, Senegal, and Mali), two from East Africa (Tanzania and Kenya) and two from Southern Africa (Malawi and Mozambique). The Malawi assessment used the ECSAT only, the Kenya one used a combination of the ECSAT and the EHSA while the other six assessments had started before the ECSAT was available and used the EHSA tool as a guidance. The assessments were completed between March 2013 and November 2018 and were funded by either Sightsavers or by the UK Department for International Development (DFID) through Sightsavers. All assessments were led by the national eye health coordinators and received technical support from consultants appointed by either the Ministry of Health or jointly by the Ministry of Health and the funder (Table 1).

**Table 1:** Assessments included in the review

Study title	Month/year completed	Tool used	Funder	Technical assistance
Sierra Leone Eye Health System Assessment	March 2013	EHSA	Sightsavers	ICEH/LSHTM
Ghana Eye Health System Assessment	March 2013	EHSA	Sightsavers	ICEH/LSHTM
Tanzania Eye Health System Assessment	May 2017	EHSA	Sightsavers	Sightsavers
Malawi Eye Care Service Assessment	May 2017	ECSAT	Sightsavers	Sightsavers
Kenya Eye Health System Assessment	November 2017	EHSA/ECSAT	UK DFID	Sightsavers
Mali Eye Health System Assessment	November 2017	EHSA	Sightsavers	Sightsavers
Mozambique Eye Health System Assessment	November 2018	EHSA	UK DFID	Sightsavers
Senegal Eye Health System Assessment	July 2018	EHSA	Irish Aid	Sightsavers

### Eye health governance

All eight assessments reported considerable progress in integrating eye care services within the broader health system, particularly at the national level. All eight countries had set up eye health programmes and the majority had up to date national eye health plans, which were in line with the global strategy for universal eye health coverage and cascaded from the broader national health policies. Mali and Senegal were the only countries with expired national eye health plans at the time of the assessment.

All countries had national multi-sectoral bodies, which aimed to coordinate eye care activities between the government, NGO, and private sector stakeholders. In all countries, the eye health coordination units were set up under the Ministries of Health and all national level regulations for eye health were the same as for the rest of the sector. These coordination and management structures however were often perceived to be weak due to the lack of influence within the Ministry of Health and limited financial and human resources to carry out the activities. There were also issues with the coordination in the country settings where eye health fell under the remit of more than one department in the Ministry of Health, for example curative and preventive services.

At the regional and district levels, the integration of eye health within the broader health systems varied and depended on the availability of eye health infrastructure, personnel, and financial resources. Overall, local coordination structures, such as Vision 2020 committees, were often either non-existent or latent. The majority of studied countries had local Disabled Persons Organisations (DPOs) involved in eye care policies and often in some aspects of service delivery. Their remit and influence, however, varied from country to country. In some settings, their presence was limited to the regional and district levels and their involvement in planning and resource allocation was minimal [22, 23]. At the time of the assessment, Senegal and Mozambique were the only two countries where DPOs were not involved in strategic planning and decision-making with regard to eye health and there was no institutional framework for their participation in policy-making [23, 24].

### Eye health financing

The countries reported a range of sources of funding for eye care, including the government, international donors, insurance schemes and out-of-pocket payments. However, none of the assessments provided accurate accounts of eye health expenditures at either national or local levels. All assessments reported that the figures were not available and at best, referred to the estimates made by national eye health coordinators [22–29].

Overall, the resources allocated to eye health by the governments were thought to be very limited, well below the level required to meet population needs [22, 24–29]. None of the countries had government allocations specific for eye health. National budgets were allocated to the departments within which the eye care unit was located; regional and district funding was allocated to all health services provided by the facilities. As a result, eye health had to compete with other priority diseases and was often overlooked, as eye conditions were not life threatening and the resources for eye care were often available through iNGOs and Faith-Based Organizations (FBOs). Where the resources were provided by the government, they covered staff salaries and facility infrastructure with little or no allocation to equipment or consumables.

Inclusion of eye care in the national health insurance schemes was reported in seven countries but there was little information on how effective these schemes were. A common view was that the schemes covered only a small proportion of the population, largely civil servants or those in formal employment, and many eye care services were not included in the package. As a result, external sources and user fees were often perceived as the main sources of funding for eye care, although not all countries allowed charging user fees within the government facilities. User fees, where reported, varied considerably between geographic areas and facilities but were generally high and beyond the reach of many patients. Four countries reported exemptions and fee waivers for vulnerable populations, but there was no information on how these schemes worked in practice [23–25, 29]. In Senegal, participants mentioned that the government supported user-fee exemptions through a system managed by the social service department at each hospital, who set a criterion for providing subsidies and assessed patients for eligibility.

### Eye care service delivery

Not all assessments reported the exact number of eye care facilities available. Overall, a range of facilities were described at different tiers of the system with significant variations in services provided dependent upon the facility level, available space and equipment and eye care skills.

Primary eye care (PEC) services were mentioned in all assessments, but their availability was dependent on whether primary care staff were trained in eye care and whether they had the necessary equipment and supplies. Where PEC services were provided, they were often limited to health education, basic eye examination and treatment of simple eye conditions. Uptake of referrals for more complex cases was mentioned as a key problem at this level.

The availability of secondary level facilities varied between and within the countries, and tertiary facilities were located mainly in the capital and a few large urban centres. Outreach eye care services were thought to be important in all countries, but these were often dependent on the funding available from iNGOs.

Service outputs reported in the assessments also varied. Cataract Surgical Rate (CSR) ranged from 494 per million in Kenya (2014) to 1343 per million in Mali (2015) and was well below the recommended levels for Africa (2000 per million in 2010) in all eight country settings. Access to refractive error and low-vision services was limited. The lack of routine monitoring of quality of cataract surgeries and services more broadly was also a challenge.

### Human resources for eye health (HReH)

The majority of the countries had national Human Resources for Health (HRH) plans with eye care staffing levels integrated within them. All countries had training institutions for specialist eye care cadres and most eye care workers were deployed by the government in the public sector. However, the number of ophthalmologists/surgeons available in the countries at the time of the assessments varied from 1.1 per million in Malawi to 4.3 per million in Kenya; and all countries, except Kenya and Senegal, had the number of surgeons well below the recommended level for Africa (4 per million). The number of mid-level ophthalmic personnel also varied from 3.0 per million in Kenya to 13.7 per million in Ghana and was below the recommended level (10 per million) everywhere except Ghana.

All countries reported unequal distribution of eye care workers, particularly ophthalmologists with between 49 and 88% of them being based within the capital city. The number of optometrists was very small and most of them were deployed in the private sector. There were concerns and ambiguity over the training and deployment of optometrists and cataract surgeons across most countries, as there were no regulations, or the regulations were contested on professional reverence grounds. Although the scope of optometry is clear in most assessed countries, in Mozambique the current optometry curriculum is still unclear. The role of general health workers in eye care was not clearly defined in most country settings.

### Medicines, products, and equipment

In all eight countries, medical products and medicines for eye health were included in the national essential medicine lists and the procurement regulations for the public sector were the same as for other health services. However, the resources available to the government were enough to cover only a fraction of supplies needed and international donors and iNGOs were reported to provide essential equipment and consumables to selected facilities and regions. It was also reported that there were no specific budget allocations for eye equipment and medicines, and they tended to be given lower priority in the public procurement systems.

The availability of medicines and functionality of equipment in the facilities surveyed in the assessments varied with some facilities having functional procurement and maintenance systems and others reporting them to be erratic. Many eye care staff were reported to be operating with no more than the basic instruments and stock-outs of essential medicines were common in most settings. The lack of proper equipment and medicines impacted negatively on staff morale and ability to keep practical skills up to date.

### Health management information system (HMIS)

Majority of countries indicated that eye health indicators were collected as part of the integrated national health information systems and there were standardised forms for recording data. In Mozambique however, eye health is not an integral part of the national health system information. Most countries had designated focal persons for reporting data; but data collection at the lower levels was manual and many data entry errors were reported.

The number of eye health indicators collected varied between the countries. International non-governmental organizations tended to collect a wider range of indicators than the national systems, but this information was limited to specific diseases (e.g. Neglected Tropical Diseases (NTDs)) or regions. The availability of population-based data on prevalence and causes of visual impairment was also limited to selected districts where iNGOs supported Rapid Assessments of Avoidable Blindness (RAABs).

There were reported delays in passing information within the HMIS and limited capacities to collect and analyse the data at all levels, which results in incomplete data, thus impacting on its usefulness. National eye health coordinators often did not have the most up to date statistics and there were no annual reports with an overview of projects implemented throughout the country. Private facilities were reluctant to report their data in all settings.

## Discussion and conclusions

To the best of our knowledge, this is the first meta-synthesis to explore and attempt to learn from assessments that document the strengths and weakness of eye care delivery in sub-Saharan Africa countries. We found marked strengths and improvements in the governance, financing, organisation, and provision of eye care services across the countries included in this review. Significant progress has been made in developing national governance frameworks, co-ordination structures, and regulatory mechanisms aligned with the rest of the sector. Even in some of the poorest countries, our meta-synthesis revealed that governments had made noteworthy efforts in taking over the responsibility for funding eye care infrastructure and salaries of eye care personnel. Similarly, important steps have been made in strengthening multi-sectoral collaboration and partnerships with disabled persons organisations.

However, despite these significant improvements, a range of systemwide weaknesses continue to impede the performace of eye health systems across the sub-Saharan Africa settings we reviewed and prevent them from achieving better results. Low per capita spending on health coupled with the historically vertical nature of eye care funding and delivery through private for profit and not-for profit providers continue to limit meaningful integration of eye care within the broader healthcare systems, particularly at the local level, where most of the service delivery takes place. As is true of most countries in Africa, the limited number of health workers in general and eye care workers in particular, continues to be a major problem creating service delivery bottlenecks at all levels. Although most of the countries attempted to develop pre-payment schemes and exemptions for vulnerable populations, the actual mechanisms of how these schemes work and their effectiveness for achieving universal health coverage remain unclear.

In addition, this review found that there was a lack of detail available across the different blocks of the health system. There are interrelated operational and broader health system causes for this lack of detail. Information and issues may be known to some actors in the system, but often there is no proper documentation or records management thus making over-reliance on desk-review as a data collection method largely ineffective. The ECSAT appear to have been designed on the assumption that the information is out there, documented – and that somebody only needed to conduct a desk review, but we found that not to be the case. The EHSA tool attempts to go around this problem by incorporating a heavy focus on primary fieldwork through face to face interviews with relevant stakeholders but the people who would be best positioned to answer questions tend to be ‘too busy’ and rarely make themselves available for an in-depth interview. However, our findings in no way imply that that government officials are reluctant to share information. Rather, the findings point to an urgent need for enhanced government efforts to strengthen internal mechanisms, infrastructure, and capacity to support and enable timely flow and sharing of information. Data are collected and analysed within vertical programmes but often reports are not distributed widely among the relevant government departments or partners. Sightsavers approach has been to try to work in collaboration with governments to make these eye health system assessment reports available online and provide some assistance for the countries included in this review to be able to host the assessment reports on MOH websites. Additionally, there is a page for the ECSAT tool on the WHO website – presumably, a link to the reports could be possible and make the reports more widely available.

The challenges identified in this review are not new and have been previously documented [14, 30, 31]. But the two tools used to guide the assessments included here provide new opportunities for reflection and rethinking of how best to work within *struggling* health systems to foster the necessary changes that would assure constant improvements in the organization and delivery of vision and eye health services. Meaningful progress toward the VISION 2020 goals require a foundation based upon ‘health systems as learning systems’, data synthesis and use [32]. In line with Blanchet et al. [11, 14, 31, 33], who have previously advocated for system thinking in eye health, we would encourage national governments and iNGOs to invest in conducting and wider use of these assessments. Such analyses are particularly useful in building links between different system elements and in finding innovative, more flexible solutions and partnerships, which can ultimately help to eliminate avoidable visual impairment in resource-poor settings.

It does feel that there should be some further discussion about the usefulness of the two tools, however, we had only one ECSAT report therefore such a discussion appears to be beyond the scope of this study. We do not claim that one tool is better than the other, but given the limited documentation and poor records management within government departments in the region, a tool that prioritizes primary fieldwork through in-depth interviews with the different actors would seem best suited in these settings.

A few potential limitations must be considered when interpreting our findings. First, the focus on assessments conducted in sub-Saharan Africa precludes the generalizability of our findings to other parts of the world. Second, despite the comprehensive searches conducted and informal discussions in-country with the national eye care coordinators, we cannot be sure that we captured all the relevant studies as we did not formally contact MOH or WHO to identify the existence of unpublished reports. Third, the EHSA/ECSAT methodology uses primarily qualitative inquiry with purposively selected stakeholders – and the tools were designed to collect and report evidence in a format relevant to decision-making in a particular context. It would therefore be difficult to compare them against one unified tool for quality assessment.

## Data Availability

All data generated or analysed during this study are included in this published article.

## Abbreviations

EHSA: Eye Health System Assessment
ECSAT: Eye Care Service Assessment Tool
MOH: Ministry of Health
WHO: World Health Organization
iNGOs: International Non-governmental Organizations
RAAB: Rapid Assessment of Avoidable Blindness
NTDs: Neglected tropical diseases
HMIS: Health Management Information System
HRH: Human Resources for Health
HReH: Human Resources for Eye Health
CSR: Cataract surgical rate
FBO: Faith based Organization
PEC: Primary eye care
NGO: Non-governmental Organization
DPOs: Disabled Persons Organisations
LSHTM: London School of Hygiene and Tropical Medicine
ICEH: International Centre for Eye Health
DFID: Department for International Development
IRB: Institutional Review Board
SSA: Sub-Saharan Africa
